# Invasive Mussels Alter the Littoral Food Web of a Large Lake: Stable Isotopes Reveal Drastic Shifts in Sources and Flow of Energy

**DOI:** 10.1371/journal.pone.0051249

**Published:** 2012-12-17

**Authors:** Ted Ozersky, David O. Evans, David R. Barton

**Affiliations:** 1 Ontario Ministry of Natural Resources, Trent University, Peterborough, Ontario, Canada; 2 Department of Biology, University of Waterloo, Waterloo, Ontario, Canada; Northwestern University, United States of America

## Abstract

We investigated how establishment of invasive dreissenid mussels impacted the structure and energy sources of the littoral benthic food web of a large temperate lake. We combined information about pre- and postdreissenid abundance, biomass, and secondary production of the littoral benthos with results of carbon and nitrogen stable isotope analysis of archival (predreissenid) and recent (postdreissenid) samples of all common benthic taxa. This approach enabled us to determine the importance of benthic and sestonic carbon to the littoral food web before, and more than a decade after dreissenid establishment. Long term dreissenid presence was associated with a 32-fold increase in abundance, 6-fold increase in biomass, and 14-fold increase in secondary production of the littoral benthos. Dreissenids comprised a large portion of the post-invasion benthos, making up 13, 38, and 56% of total abundance, biomass, and secondary production, respectively. The predreissenid food web was supported primarily by benthic primary production, while sestonic material was relatively more important to the postdreissenid food web. The absolute importance of both sestonic material and benthic primary production to the littoral benthos increased considerably following dreissenid establishment. Our results show drastic alterations to food web structure and suggest that dreissenid mussels redirect energy and material from the water column to the littoral benthos both through biodeposition of sestonic material as well as stimulation of benthic primary production.

## Introduction

The establishment of invasive organisms is a threat facing aquatic ecosystems world wide [1]–[3]. Among the most significant–but often most difficult to quantify–ecological impacts of species invasions are alterations to food web structure and energy flow patterns [3]. Stable isotope analysis (SIA) of carbon and nitrogen in constituents of the food web offers a way to untangle the complex effects of species invasions and other perturbations on the trophic structure of ecosystems. Carbon isotope ratios are generally conserved between food sources and consumers, so ^13^C/^12^C ratios can be used to determine the contribution of different energy sources to consumer diets [4]–[6]. Nitrogen isotopic composition changes predictably with movement up the food web, allowing the determination of consumer trophic level if the nitrogen isotopic composition of basal resources in known [6], [7]. Together, C and N stable isotopes have been used to characterize the effects of invasive organisms on the flow of energy through ecosystems [8], [9], food web length [10], and resource partitioning [11].

Few aquatic invaders have received as much attention from ecologists as the zebra and quagga mussels (*Dreissena polymorpha* and *D. rostriformis bugensis*, respectively). Yet, despite decades of study, the effects of dreissenids on energy flow and food web structure are not fully understood. Dreissenids are increasingly seen as habitat-couplers, redirecting nutrients, energy, and production from the water column to the littoral benthic region of lakes [12]–[14]. Dreissenids are filter-feeders that graze on seston and release undigested and unwanted particles as feces and pseudofeces (material collectively known as biodeposits) on the lake bottom, thereby increasing the flux of sestonic material to the benthos [15], [16]. By clearing the water column and excreting dissolved nutrients at the substrate-water interface dreissenids also increase light penetration and nutrient availability in the nearshore, stimulating primary production of benthic algae [13], [17]. Thus, dreissenids can compete with other filter-feeders for seston while increasing resource availability for littoral detritivores, grazers, and predators. Studies of the effect of dreissenids on benthic communities indeed show declines in the abundance of native filter feeders, and increased abundance of detritivores, some grazers, and benthic predators following dreissenid establishment [14], [18], [19].

In addition to evidence from studies of community structure, SIA approaches have shed some light on the way dreissenid establishment affects food webs. Because seston is often depleted in ^13^C relative to benthic algae [5], [20], carbon isotope ratios can be used to determine how dreissenids affect the importance and availability of these two energy sources to food webs. SIA of carbon has been used to show that dreissenids can compete with native filter feeders for seston [21], [22], and that redirected sestonic material in the form of dreissenid biodeposits can contribute significantly to the diet and production of detritivores such as amphipods and chironomids [11], [23]. However, there have been no comprehensive examinations of how dreissenid establishment affects the absolute importance of benthic primary production to food webs, or the relative balance between sestonic and benthic energy sources.

The littoral zone can dominate production processes and the transfer of energy to higher trophic levels in lakes [5], [24], making it important we understand how perturbations affect the food web structure and energy dynamics of littoral ecosystems. To investigate the effect of dreissenids on the littoral food web of a large lake we compared the abundance, biomass, and production of hard substrate-inhabiting littoral benthos collected prior to, and more than a decade following dreissenid establishment in Lake Simcoe, Ontario. We performed SIA of all common members of the pre- and postdreissenid benthos and used isotope mixing models to estimate the importance of sestonic material and benthic primary production to the pre- and postdreissenid littoral benthic communities. Our objectives, by combining information about the biomass, production, and energy sources of different faunal groups, were to i) determine whether dreissenid establishment has had an impact on food web structure of the littoral benthos and, ii) quantify the impact of dreissenid establishment on the relative and absolute importance of sestonic and benthic energy sources to sustaining the littoral benthic food web.

## Methods

### Ethics Statement

No specific permits were required for the described field studies. All field studies were carried out on public property and did not involve protected or endangered species.

### Study Site

**Figure 1:**
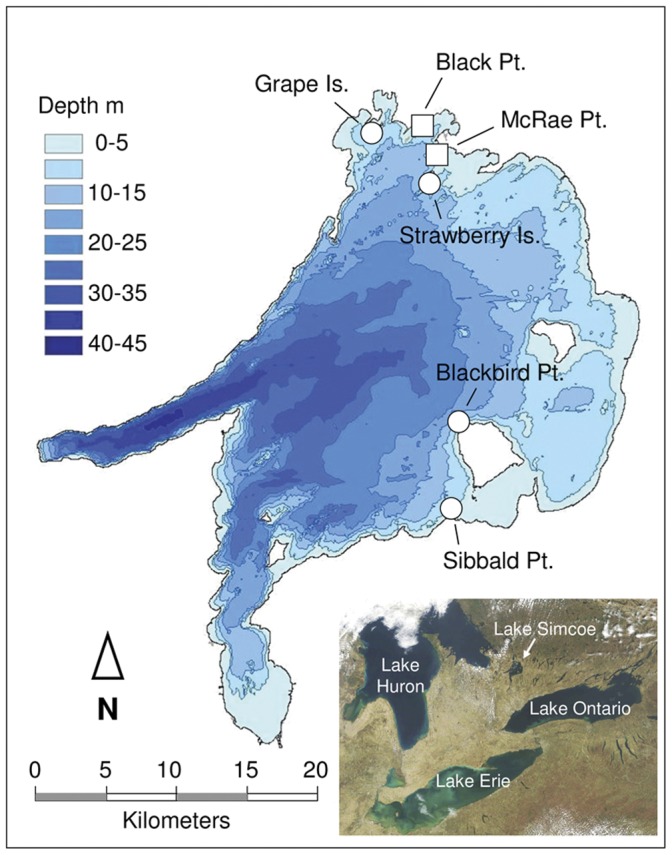
Map showing the locations of sampling sites. Circles represent sites where quantitative benthos samples were collected, squares show sites where most samples for stable isotope analysis were collected.

Lake Simcoe is a large (722 km^2^), oligo-mesotrophic lake in southern Ontario, Canada (Fig. 1). The extensive littoral zone of Lake Simcoe is dominated by rocky substrates to depths of about 8 m, while soft substrates prevail at greater depths. Dreissenid mussels were first observed in the benthos of Lake Simcoe in the fall of 1994, but did not become widespread and abundant until 1996 [25].

### Sampling the Benthos, Estimating Biomass and Production

Quantitative benthic samples were collected in 1993 (predreissenid period) and in 2007–2008 (postdreissenid period), using an airlift sampler [26] operated by SCUBA divers. Fours sites (Fig. 1), selected to include mainland and island littoral habitats in different parts of the lake, were sampled in 1993 using a 0.25 m^2^ quadrat. The same locations were sampled again in 2007 for crayfish using a 0.25 m^2^ quadrat and in 2008 for other macroinvertebrates using a 0.0625 m^2^ quadrat. Three to four replicate samples were collected from 2-m depths at each site in the pre- and postdreissenid periods. Sampling in all years was carried out in late August and early to mid September.

Crayfish were stored frozen until identification, measurement, and tissue removal for SIA (see below). Quantitative samples of smaller macroinvertebrates were fixed in 10% buffered formalin and then transferred to 90% ethanol prior to enumeration and identification. Preserved airlift samples collected in 1993 from three of the four sites were counted to the order level shortly after collection and then returned to the original sample jars. Samples collected in 2008 were counted to the taxonomic level of family or lower for most groups in order to provide finer resolution for food web reconstruction. To maintain consistency of taxonomic resolution, we resorted the samples collected in 1993, then enumerated and identified invertebrates to the same taxonomic level as samples collected in 2008. Fewer animals were found in most samples during this re-sorting, which we attribute to losses caused by the original handling and processing. To estimate 1993 abundances at a taxonomic level that would allow intercomparison with the postdreissenid benthic community, we assumed that the original counts at the order level were correct and proportionally adjusted the counts for lower taxonomic levels. For example, there were 88 amphipods in the original count of replicate A from Blackbird Point. The recount found 44 *Gammarus* spp. and 12 *Hyalella azteca*, or a total 56 amphipods. We assumed that both *Gammarus* spp. and *H. azteca* suffered similar loss rates, adjusting the numbers to 69 *Gammarus* spp. and 19 *H. azteca*. We believe the assumption of equal loss across lower level taxonomic groups is reasonable given that loss rates were not significantly different across orders (Kruskal-Wallis one-way analysis of variance, *p*>0.05).

Dry biomass of most organisms was estimated from site-specific, length-dry weight relationships (Text S1) and observed, period-specific size-frequency distributions. In cases where we did not have sufficient material to construct site-specific length-dry weight relationships, published relationships were used (references in Text S1). Annual production of benthic invertebrates was estimated using the empirical model of Morin and Bourassa [27], which uses mean areal biomass, mean individual biomass, and mean annual water temperature to estimate taxon-specific secondary production. We report the abundance, biomass and production only for taxa that were relatively abundant (>5% of total abundance) in either the pre- or postdreissenid period, or that were deemed to contribute greatly to biomass and production because of their large individual size (e.g., crayfish). For a complete listing of invertebrate taxa at these sites see [19].

### Collection and Processing of Samples for SIA

Seston for SIA was collected along the south-eastern shoreline of the lake from 8 sites of ∼1 m depth in July and August of 2008. Two 500 ml PET jars were submerged to a depth of ∼0.5 m, filled with lake water and placed on ice. In the lab seston samples were filtered onto precombusted quartz fibre filters, dried at 60°C for 24 hours and stored in a dessicator until preparation for isotope analysis. Periphyton scrapes were collected by a snorkeller from rocks at ∼1–1.5 m depth at McRae Point in September of 2008 (Fig. 1). A 60 ml syringe with an 18-gauge needle was used by a snorkeller to collect biodeposits and detritus from beneath mussel colonies. We observed mussels expelling fresh biodeposits during our sampling and gently collected this material using a syringe fitted with a 10 cm length of 2-mm Ø Tygon® tubing. In the lab biodeposit samples were filtered onto precombusted quartz fibre filters, dried at 60°C for 24 hours and stored in a glass dessicator until SIA. Samples collected from the surface and from beneath mussel colonies had similar stable isotope values, so these data were combined and are reported together.

Most small macroinvertebrates used for SIA were collected in September of 1993 and 2008 from shallow depths (∼1 m) at McRae Point using D-nets with 500-µm nitex mesh and kick and sweep sampling. Macroinvertebrates were separated into general taxonomic groups, allowed to empty their guts for 24 hours in refrigerated containers with lake water, and stored frozen in de-ionized water until analysis. We did not have sufficient frozen material from McRae Point to cover all taxonomic groups, so frozen samples from the predreissenid period were supplemented with formalin-preserved invertebrates collected at McRae Point and adjacent Black Point at 2 m depth in the fall of 1993. SIA results of formalin-preserved samples were corrected for the effect of long-term formalin preservation, which was shown to result in a 2‰ depletion to δ^13^C values and no significant change to δ^15^N values of aquatic invertebrate tissues [28].

Our samples from 2008 were supplemented with specimens of common but less abundant invertebrates collected by kick and sweep sampling at McRae Point and Black Point in September of 2009. Where sufficient numbers of samples of the same taxa were available for a statistical comparison of isotope values of frozen material from 2008 versus 2009, and frozen versus formalin-preserved (and preservation effect-corrected) material from 1993 no significant differences in ^13^C values were found, and only small (<1‰) differences in ^15^N values were seen in the case of postdreissenid *Gammarus* sp. and chironomids (two-sample independent t-tests at *α* = 0.05); we therefore combined the data. Crayfish for SIA were collected from depths of 2, 4, and 6 m at two sites adjacent to McRae Point (Strawberry Island and Grape Island, Fig. 1). A comparison of the results showed no statistically significant difference in crayfish isotopic composition with depth or between sites (one-way ANOVAs, *p*>0.05), so crayfish from all depths were pooled for analysis.

Samples of periphyton, crayfish dorsal abdominal muscle, and other invertebrates were prepared for SIA by drying for 24–48 hours at 60°C. Animals other than crayfish were either ground whole or after removal of shells in the case of gastropods and bivalves. When necessary to achieve a minimum sample weight of 0.25 mg, individuals within taxa were combined into a single isotope sample. Biodeposit samples and seston samples were analysed on quartz filters. Seston and biodeposit samples were split into two portions, one of which was acidified by fumigation in a glass dessicator with concentrated hydrochloric acid to remove carbonates [29]. Periphyton samples were acidified in glass vials by slowly adding 10% HCl until visible bubbling stopped, and were then redried, ground and weighed. Samples were analyzed on a Delta Plus continuous flow mass spectrometer (Thermo Finnigan, Germany) coupled to Carlo Erba elemental analyzer (CHNS-O EA1108, Italy) at the University of Waterloo Environmental Isotope Laboratory. Replicate samples had a standard deviation of 0.21‰ for ^13^C and 0.28‰ for ^15^N.

### End-member Selection, Mixing Models

**Figure 2:**
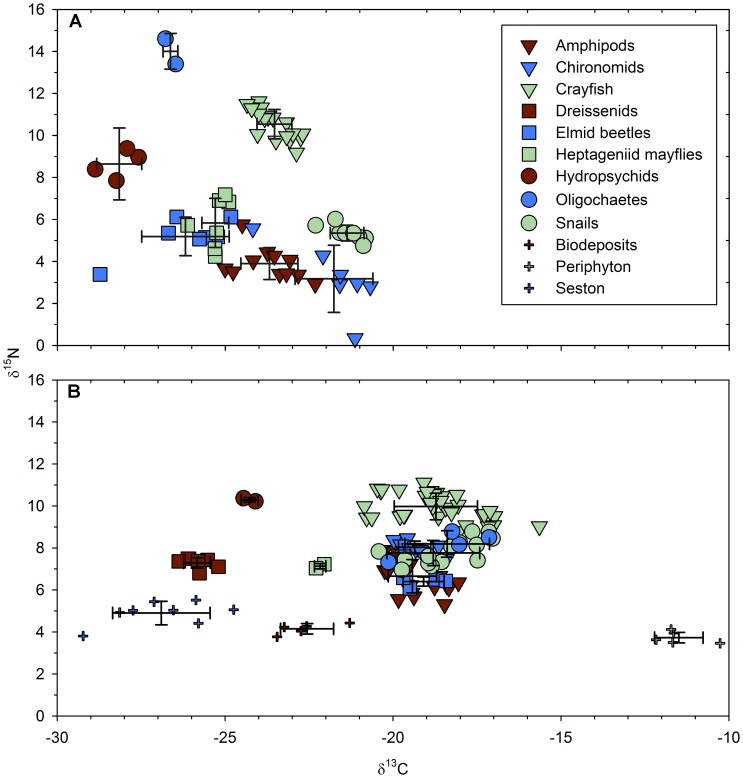
δ^13^C and δ^15^N isotope biplots for members of the littoral food web of Lake Simcoe. A) Predreissenid period. B) Postdreissenid period. Only taxa with more than one replicate that were either sampled in both periods, or contribute more than 5% to pre- or postdreissenid biomass or production are shown. Error bars represent one standard deviation.

A two-source, single-isotope (^13^C) linear mixing model (IsoError [30]) was used to estimate the relative importance of sestonic material and benthic primary production to taxa in the pre- and postdreissenid periods. No primary producers were available from the 1993 collections. Consequently snails, which are considered to be periphyton grazers [6], were used as the benthic end-member and filter-feeding hydropsychid caddisflies [31] as the sestonic end-member for the predreissenid mixing model. For the postdreissenid period we examined the effect of using two combinations of end-members on food web reconstruction. In our first approach we used seston and periphyton as the sestonic and benthic end-members (seston-periphyton model). In our second approach we used primary consumers as end-members, reasoning that since primary consumers were used as end-members for the predreissenid period, a postdreissenid mixing model based on primary consumers would enable a more direct comparison between periods. We used dreissenids as the sestonic end-member and psephenid beetle larvae (water pennies) as the benthic end-member because dreissenids appear to rely almost exclusively on seston, while psephenids are known to be grazers of periphyton [31], [32] and were the closest to periphyton in ^13^C values among postdreissenid consumers (dreissenid-psephenid model).We decided not to use snails as the benthic end member in our postdreissenid reconstruction because snails were no longer the most enriched member of the fauna (Fig. 2), suggesting that they incorporated more sestonic material than snails in the predreissenid period. The percent contribution of sestonic and benthic material for each taxon obtained from the mixing models together with taxon-specific biomass and production estimates were used to determine the relative and absolute contribution of benthic and sestonic resources to the nearshore food web in the pre- and postdreissenid periods.

### Assumptions

We did not perform SIA on sphaeriid clams, assuming 100% reliance on pelagic carbon. While the literature partially supports this assumption (e.g., [33], [34] and references therein), overestimation of the contribution of sestonic material to sphaeriids should not greatly affect overall conclusions because sphaeriids comprised only a small fraction of pre- (0.09 and 1.11%) and postdreissenid (0.02 and 0.13%) biomass and production, respectively. We also did not perform SIA on the crayfish *Orconectes virilis*, but results from other locations in Lake Simcoe show that *O. virilis* has similar δ^13^C and δ^15^N values to *O. rusticus* and *O. propinquus* (DO Evans, unpublished data). We assigned the isotopic value of *O. virilis* based on the average isotopic values of 5 randomly selected *O. rusticus* and 5 *O. propinquus* from our dataset. Only one sample of the amphipod *Crangonyx* sp. from 1993 was analysed; the average standard deviation of δ^13^C values of two other amphipods (*Gammarus* spp. and *H. azteca*) was used to approximate the standard deviation of δ^13^C values of *Crangonyx* sp. for use in our mixing model.

## Results

### Abundance, Biomass, Estimated Production

Abundance, biomass, and estimated production (EP) of the nearshore benthos were much higher in 2007–2008 than in 1993, the year immediately preceding dreissenid establishment in Lake Simcoe. The abundances of most individual taxa as well as total invertebrate abundance were significantly greater in the postdreissenid period (Table 1; Wilcoxon rank-sum tests at *α* = 0.05). Total invertebrate abundance increased almost 33-fold from an average of 795.2 (±126.2 SE) to 25,807.0 (±1885.3 SE) individuals m^−2^, total biomass increased approximately 6-fold from 10.7 to 60.3 g dry wt m^−2^, and total EP increased 14-fold from 6.4 to 90.9 g dry wt m^−2^ year^−1^ (Table 1). Dreissenids contributed to much of the increases in biomass and EP of the benthos, constituting 37.7% of the total biomass and 55.8% of total EP in the postdreissenid period (Table 1). Two recent invaders, the crayfish *O. rusticus* and the amphipod *Echinogammarus ischnus*, together comprised 33% of non-dreissenid biomass and 19.7% of non-dreissenid EP in 2007–2008 (Table 1).

**Table 1:** Mean abundance (standard error in parentheses), biomass, and annual production of benthic organisms in pre- and postdreissenid periods from 4 shallow (2 m) sites in Lake Simcoe.

Period:	Predreissenid			Postdreissenid		
Taxon	Abundancem^−2^	Biomassmg m^−2^	Productionmg m^−2^ yr^−1^	Abundancem^−2^	Biomassmg m^−2^	Productionmg m^−2^ yr^−1^
Amphipoda						
* Hyalella azteca*	117.1 (30.9)	11	92	4414.9 (882.1)*	313	3280
* Gammarus* spp.	11.7 (4.1)	3.7	18	3820.6 (749.2)*	1020	6850
* Echinogammarus ischnus*	not present	not present	not present	2652.6 (691.3)	446	3500
* Crangonyx* sp.	6.2 (2.3)	0.70	4.6	1146.3 (408.6)*	143	1230
Isopoda						
* Caecitodea racovitzai*	0.0	0	0	1516.6 (366.2)*	385	2620
Decapoda						
* Orconectes propinquus*	17.6 (2.8)	9970	4870	19.0 (4.4)	17930	7710
* Orconectes rusticus*	not present	not present	not present	10.8 (4.8)	11950	4420
* Orconectes virilis*	0.0	0	0	1.5 (0.7)*	4040	1160
Gastropoda						
Physidae	15.9 (6.0)	76	154	51.4 (18.9)	142	427
Hydrobiidae	19.7 (6.3)	3.7	22	278.9 (88.6)*	89	553
Pleuroceridae	26.2 (8.5)*	520	699	0.0	0	0
Bivalvia						
* Dreissena* spp.	not present	not present	not present	3366.3 (688.3)	22720	50760
Sphaeriidae	74.3 (12.7)	14	84	146.3 (45.6)	12	114
Insecta						
Chironomidae	296.8 (112.0)	12	124	3709.7 (698.4)*	248	2640
Polycentropodidae	17.6 (4.4)	13	45	171.4 (46.8)*	104	519
Heptageniidae	46.8 (8.3)	22	104	115.4 (48.1)	42	247
Elmidae	45.2 (11.0)	9.2	60	227.7 (135.0)	49	372
Oligochaeta	98.1 (43.1)	16	93	3798.9 (653.6)*	565	372
Platyhelminthes	2.0 (1.3)	0.60	3.0	358.9 (120.3)*	107	4170
**All taxa combined**	**795.2 (126.2)**	**10670**	**6370**	25807.0 (1885.3)*	**60300**	**90900**

Asterisks appear beside the significantly larger number as determined by Wilcoxon rank-sum test at α = 0.05.

The composition of the benthic community shifted considerably after dreissenid establishment. The predreissenid community was numerically dominated by chironomids and amphipods, while crayfish, snails, mayflies, oligochaetes and amphipods accounted for the bulk of biomass and EP (Table 1). Much of the enhanced postdreissenid abundance was due to large increases in the abundances of amphipods, isopods, chironomids, oligochaetes, and the introduction of dreissenids. Crayfish (33.9 g m^−2^) and dreissenids (22.7 g m^−2^) dominated the postdreissenid biomass, with amphipods, oligochaetes, and isopods also contributing considerably to total biomass. Dreissenids accounted for more than half of EP in the postdreissenid period, followed by small crustaceans, i.e. amphipods and isopods (19.2%), crayfish (14.6%), flatworms (4.6%), and chironomids and other Insecta (4.2%) (Table 1).

### SIA Results

Snails were the most enriched in ^13^C among the predreissenid benthic taxa (−21.4±0.5 SD ‰), and filter-feeding hydropsychid caddisflies the most depleted (−27.9±0.7 SD ‰). Chironomids were the most depleted group in ^15^N (3.2±1.2 SD ‰), and large lumbriculid oligochaetes the most enriched (14.0±0.85 SD ‰) (Fig. 2A, Table 2).

**Table 2:** Summary of results of ^13^C and ^15^N isotope analysis (standard deviation in brackets) for taxa collected in the pre- (1993) and postdreissenid periods (2007–2009).

Group	Year of collection	Preservation method	*n*	Average δ^13^C	Average δ^15^N
Chironomidae	1993	formalin	7	−21.76 (1.16)	3.17 (1.60)
*Crangonyx* sp.	1993	formalin	1	−25.55	10.35
Elmidae	1993	frozen (1), formalin (6)	7	−26.19 (1.30)	5.19 (0.92)
*Gammarus* spp.	1993	frozen (5), formalin (1)	6	−23.96 (1.01)	3.95 (1.01)
Heptageniidae	1993	frozen (4), formalin (3)	7	−25.30 (0.40)	5.83 (1.17)
*Hyalella azteca*	1993	formalin	5	−23.36 (0.52)	3.83 (0.41)
Hydropsychidae	1993	frozen	5	−27.94 (0.67)	7.92 (1.71)
*Orconectes propinquus*	1993	frozen	18	−23.54 (0.52)	10.54 (0.70)
Oligochaeta	1993	frozen	2	−26.63 (0.22)	14.00 (0.85)
Snails	1993	frozen	8	−21.37 (0.50)	5.35 (0.38)
biodeposits	2008	frozen	6	−22.56 (0.79)	4.15 (0.25)
periphyton	2008	frozen	5	−11.49 (0.72)	3.73 (0.29)
seston	2008	frozen	8	−26.90 (1.45)	4.90 (0.56)
Chironomidae	2008 (4), 2009 (7)	frozen	11	−19.36 (0.48)	8.03 (0.30)
*Dreissena* spp.	2008	frozen	7	−25.84 (0.40)	7.29 (0.25)
*Echinogammarus ischnus*	2008	frozen	6	−18.60 (0.45)	6.05 (0.48)
Elmidae	2008	frozen	4	−19.09 (0.61)	6.40 (0.22)
*Gammarus* spp.	2008 (7), 2009 (2)	frozen	9	−19.66 (0.55)	7.09 (0.78)
Heptageniidae	2008	frozen	2	−22.17 (0.17)	7.13 (0.13)
*Hyalella azteca*	2009	frozen	5	−19.99 (0.27)	6.59 (0.71)
Hydropsychidae	2008	frozen	2	−24.27 (0.25)	10.29 (0.11)
Isopoda	2008 (6), 2009 (1)	frozen	7	−19.23 (0.27)	6.54 (0.52)
*Orconectes propinquus*	2007	frozen	17	−18.49 (0.58)	10.29 (0.41)
*Orconectes rusticus*	2007	frozen	18	−18.94 (1.63)	9.68 (0.67)
Oligochaeta	2009	frozen	4	−18.39 (1.26)	8.19 (0.63)
Platyhelminthes	2008	frozen	4	−20.54 (0.39)	11.55 (0.54)
Polycentropodidae	2008	frozen	4	−21.08 (0.72)	9.77 (0.23)
Psephenidae	2008	frozen	5	−15.82 (1.57)	5.65 (0.28)
Snails	2008	frozen	14	−18.44 (1.04)	7.82 (0.60)

Number in brackets beside year of collection and preservation method indicates how many of the total number of samples (*n*) were collected in that year or preserved using that method for taxa for which samples from more than one year or preservation methods were used.

Postdreissenid δ^13^C values of all benthic taxa were bracketed by seston (−26.9±1.45 SD ‰) and periphyton (−11.5±0.7 SD ‰). Dreissenid biodeposits were found to be more enriched in δ^13^C than seston (−22.6±0.8 SD ‰), suggesting the possibility of inclusion of periphyton or macrophyte detritus in our biodeposit samples, preferential rejection by dreissenids of this ^13^C enriched fraction from their filtered intake, or enrichment during metabolic processing by mussels or bacteria. The most ^13^C enriched members of the post-dreissenid fauna were psephenid beetle larvae (−15.8±1.6 SD ‰ δ^13^C), and dreissenid mussels were the most depleted (−25.8±0.4 SD ‰ δ^13^C). Psephenid beetles (5.7±0.3 SD ‰ δ^15^N) and flatworms (11.6±0.5 SD ‰ δ^15^N) were the most depleted and enriched faunal groups in ^15^N, respectively (Table 2).

The nine groups that were sampled in both the pre- and postdreissenid periods, and had sufficient replicates for a statistical comparison (*Gammarus* spp., *Hyalella azteca*, *O. propinquus, Physa* sp., Chironomidae, Heptageniidae, Hydropsychidae, Elmidae and Oligochaeta) were consistently and significantly more enriched in ^13^C in the postdreissenid period (two-sample independent t-tests at *α* = 0.05) by an average of 4.47 (±0.66 SE) ‰ δ^13^C (Table 2). Most of those groups also showed a significant, albeit smaller, enrichment in ^15^N (average 1.34 ‰ ±1.01 SE).

### Mixing Models

Members of the predreissenid littoral food web displayed a range of reliance on benthic and sestonic resources (Table 3). Filter-feeding hydropsychids and oligochaete worms were the most sestonic-reliant groups, and snails and chironomids the most benthic-reliant. Crayfish, which dominated the biomass and production of the predreissenid benthos were estimated to utilize 67% (±3.1 SE) benthic resources. The average, taxon-specific contribution of sestonic carbon across all taxa was 48.6%. The choice of end-members had an effect on estimates of the importance of benthic and sestonic energy sources to different taxa in the postdreissenid period. The seston-periphyton model showed a greater importance of sestonic material for most groups relative to the dreissenid-psephenid model (Table 3). The average, taxon-specific contribution of sestonic carbon across all taxa was 55.3% in the seston-periphyton model and 41.9% in the dreissenid-psephenid model (Table 3).

**Table 3:** Contribution of sestonic carbon (% sestonic) to members of the benthos in the pre- and postdreissenid periods in the shallow littoral zone of Lake Simcoe, as determined by stable isotope analysis and the IsoError isotope mixing model [30].

Period:	Predreissenid	Postdreissenid	
End-members (sestonic-benthic):	hydropsychids-snails	seston-periphyton	dreissenids-psephenids
Taxon			
Amphipods			
* Hyalella azteca*	30.3 (4.3)	55.2 (2.2)	41.6 (4.3)
* Gammarus* spp.	39.4 (6.8)	53.0 (2.3)	38.3 (4.7)
* Echinogammarus ischnus*	–	46.1 (2.3)	27.7 (5.6)
* Crangonyx* sp.	63.6 (12.1)	–	–
Isopods			
* Caecidotea racovitzai*	–	50.2 (2.1)	34.0 (4.8)
Crayfish			
* Orconectes propinquus*	33.0 (3.1)	45.4 (2.1)	26.6 (5.3)
* Orconectes rusticus*	–	48.3 (3.2)	31.1 (6.2)
Snails	0 (3.8)	47.4 (3.0)	29.7 (6.0)
Bivalves			
* Dreissena* spp.	–	93.1 (3.3)	100 (2.1)
Insects			
Chironomids	5.9 (7.1)	51.1 (2.2)	35.3 (4.8)
Polycentropodidae	–	62.2 (3.2)	52.5 (5.0)
Heptageniidae	59.8 (4.0)	69.3 (2.5)	63.4 (3.0)
Elmidae	73.4 (8.4)	49.3 (2.8)	32.6 (5.6)
Psephenidae	–	28.1 (4.9)	0 (9.9)
Hydropsychidae	100 (7.2)	82.9 (3.0)	84.3 (2.4)
Oligochaeta	80.1 (4.7)	45.1 (2.6)	26.2 (5.9)
Platyhelminthes	–	58.7 (2.5)	47.1 (4.2)

Standard error is shown in brackets.

**Figure 3:**
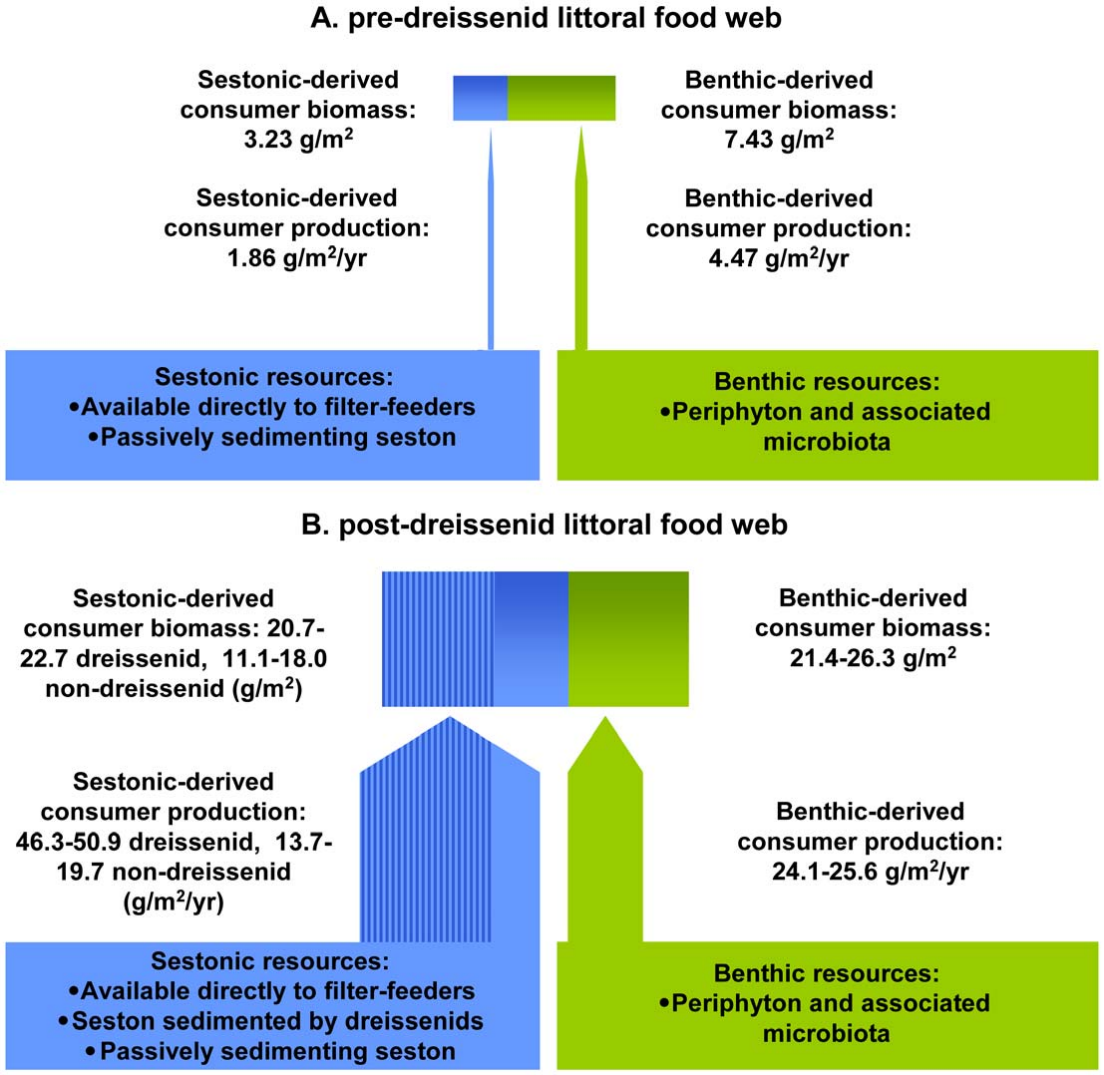
Contribution of sestonic (blue) and benthic (green) carbon to the littoral food web of Lake Simcoe. A) Predreissenid period. B) Postdreissenid period. Postdreissenid values are a range between the results of mixing models based on primary producers and primary consumers. Stippled portion represents dreissenid biomass and production.

The biomass (69.7%) and secondary production (70.6%) of the predreissenid littoral food web were supported mainly by benthic primary production (Table 4, Fig. 3), due to the domination of biomass and production by mostly benthic-reliant crayfish and snails. Most (98.5%) of the sestonic carbon in the predreissenid benthos was also contained in crayfish. Amphipods, sphaeriid clams, heptageniid mayflies, elmid beetle larvae, and worms also contributed to storage and turnover of sestonic carbon prior to dreissenid establishment (Table 3, Table 4).

**Table 4:** Amount of sestonic- and benthic-derived biomass and production in different groups of littoral benthos of Lake Simcoe, in pre- and postdreissenid periods, using hydropsychids and snails as the sestonic and benthic end-members for the predreissenid period and two combinations of end-members for the postdreissenid period.

Group	sestonic-derived biomass mg m^−2^	benthic-derived biomass mg m^−2^	sestonic -derived production mg m^−2^ yr^−1^	benthic-derived production mg m^−2^ yr^−1^
*predreissenid; end-members: hydropsychids (sestonic)- snails(benthic)*				
Small crustaceans	5	10	37	78
Crayfish	3180	6790	1550	3320
Snails	0	600	0	880
Bivalves	14	0	84	0
Insects	20	23	110	180
Worms	12	4	72	21
**All combined**	**3230**	**7430**	**1860**	**4470**
*postdreissenid; end-members: seston (sestonic)- periphyton (benthic)*				
Small crustaceans	1080	1070	8420	8270
Crayfish	16240	17600	6360	6980
Snails	100	130	440	540
Bivalves	20690	2040	46310	4570
Insects	240	200	1980	1800
Worms	310	360	2560	1980
**All combined**	**38670**	**21410**	**66070**	**24130**
*postdreissenid; end-members: dreissenids (sestonic)- psephenids (benthic)*				
Small crustaceans	770	1390	6030	10660
Crayfish	9750	24090	3810	9530
Snails	60	170	260	720
Bivalves	22730	0	50870	0
Insects	180	260	1490	2290
Worms	200	480	2060	2480
**All combined**	**33700**	**26380**	**64520**	**25680**

Contributions of sestonic material and benthic primary production to the food web shifted following dreissenid establishment. Estimates based on the seston-periphyton and dreissenid-psephenid mixing models were in agreement and revealed that, overall, sestonic material contributed more strongly than benthic primary production to the postdreissenid nearshore food web (Table 4, Fig. 3). However, estimates based on seston-periphyton and dreissenid-psephenid models provided somewhat different estimates for the importance of sestonic material versus benthic primary production to the non-dreissenid benthos. The seston-periphyton model showed that benthic and sestonic energy sources were about equally important to the non-dreissenid benthos, whereas the dreissenid-psephenid model suggested benthic primary production is about twice as important in supporting non-dreissenid biomass and secondary production (Table 4, Fig. 3).

## Discussion

The establishment of *Dreissena* spp. has resulted in dramatic changes to the structure and energy base of the littoral food web in Lake Simcoe. Long-term dreissenid presence led to large increases in the abundance, biomass, and production of littoral macroinvertebrates. We observed changes in both the relative and absolute importance of sestonic and benthic energy sources to the littoral food web, with the relative importance of sestonic material increasing following dreissenid establishment. The results of isotope mixing models and the large increases in biomass and production of the benthos imply that the absolute contribution of both sestonic and benthic material to the food web increased dramatically following dreissenid establishment (Fig. 3). These results provide evidence that dreissenids transfer energy and nutrients from the water column to the littoral benthos of lakes though deposition of sestonic material, as well as through stimulation of benthic primary production.

We found the predreissenid littoral fauna of Lake Simcoe to be typical of exposed rocky substrata in the Great Lakes region [26] with crayfish and snails dominating biomass and secondary production, and benthic primary production supporting about 70% of biomass and secondary production. The establishment of dreissenids has frequently been associated with increases in the abundance and biomass of littoral benthos, and while the increase in abundance seen in our study is among the largest reported, the qualitative changes to the benthic community generally paralleled those observed elsewhere [14], [18], with amphipods, isopods, chironomids, and oligochaetes undergoing large increases in absolute and relative abundance. It is thought that dreissenid enhancement of littoral benthos, especially on rocky substrates, results from a combination of habitat modification and increased food supply [35], [36]. Shells of living and dead dreissenids increase the availability of habitat [36] and provide refuge to invertebrates from predation by fish [37], while the increased flux of sestonic material [16], and enhanced benthic primary production rates [13], [38] provide greater food resources to benthic consumers. Until now, however, the relative and absolute importance of redirected sestonic material and benthic primary production to dreissenid-invaded food webs have not been well resolved.

Previous studies have shown that dreissenid biodeposits are used as an energy source by some littoral benthic taxa [15], [23], and our results demonstrate that redirected sestonic material forms a large portion of the energy budget of the postdreissenid littoral food web in Lake Simcoe. While sestonic carbon contributed most to the biomass and production of dreissenid mussels, it also supported between one third and half of the biomass and production of non-dreissenid benthos. The absolute importance of sestonic material to littoral biomass was about an order of magnitude greater in postdreissenid times, while the contribution of sestonic material to littoral secondary production increased by about 35-fold. Excluding dreissenids, the absolute importance of sestonic material to the biomass and production of the littoral benthos increased by between 3–5, and 7–11 fold, respectively, suggesting that dreissenid biodeposits are an important energy source to the nearshore. The notion that dreissenid biodeposits form the main source of sestonic carbon to the littoral food web is supported by the fact that water clarity increased and planktonic algal biovolumes in Lake Simcoe either decreased or remained unchanged since dreissenid establishment [39], [40], making it highly unlikely that natural sedimentation rates of sestonic material increased appreciably in postdreissenid times.

The absolute importance of benthic primary production to the benthos also increased considerably since predreissenid times. While increases in crayfish biomass accounted for much of the increase in the absolute importance of benthic carbon, all other benthic taxa (with the exception of snails) contributed to the increased utilization of benthic primary production in the postdreissenid food web. The absolute importance of benthic primary production to supporting littoral biomass approximately tripled, while its importance to supporting secondary production increased 4–6 fold. This result is consistent with observed increases in benthic primary production following dreissenid establishment [14], [38] and demonstrates that dreissenid-mediated increases in benthic primary production are translated into increased secondary production. Thus, while we saw moderate changes to the relative importance of benthic and sestonic primary production to non-dreissenid benthos, our results clearly demonstrate that the absolute importance of both increased substantially (Fig. 3).

Our choice of mixing model end-members affected estimates of the relative and absolute importance of littoral and sestonic production to the postdreissenid food web. The relative merits of the two postdreissenid mixing models we used are open to debate. On the one hand mixing models based on primary consumers are recommended because consumers average the signatures of primary producers which can vary considerably over time [6], and offer a “time-integrated” picture of the food web. On the other hand, the use of primary consumers requires assumptions about their diets. Snails are recommended as the benthic-littoral end-member in mixing models because they are assumed to feed almost exclusively on periphyton. Feeding experiments, however, show that snails often feed opportunistically (e.g., [41]), and our stable isotope results suggest that sestonic material may have comprised a considerable portion (30 to 48%) of snail diets in the postdreissenid period. The relatively high reliance of snails on sestonic material in the postdreissenid period raises the question whether snails are an appropriate benthic end-member for our reconstruction of the predreissenid food web. We believe that because sestonic material was less readily available to snails in the predreissenid period their reliance on benthic algae would have been higher than in postdreissenid times. There is still a possibility that by using snails we are overestimating the importance of benthic material to the predreissenid food web, but this is unlikely to affect the main conclusion of our study that the absolute importance of both sestonic material and benthic primary production to the littoral food web increased following dreissenid establishment.

An unexpected finding of our study was the consistent enrichment in ^13^C and ^15^N of benthic organisms in the postdreissenid period, regardless of whether the organisms were filter-feeders, detritivores, or grazers. Our mixing models show that the relative importance of isotopically-heavy benthic primary production to most taxa did not increase significantly following dreissenid establishment (Table 2), suggesting that benthic and sestonic primary producers became more enriched in ^15^N and ^13^C following dreissenid invasion. While we cannot confirm that such a shift in isotopic values of primary producers occurred, it would be consistent with increased rates of sestonic and benthic primary production often seen in dreissenid invaded systems [14]. As primary production rates increase fractionation against heavier isotopes can decrease drastically, leading to more enriched δ^13^C and δ^15^N values of benthic and sestonic primary producers [42]–[44], which is consistent with the isotopic enrichment seen in our study. The possibility that changes in primary production rates associated with dreissenid establishment and other ecological perturbations can lead to shifts in isotopic values of primary producers deserves further investigation, and may need to be considered in studies using isotopic approaches and archival samples to examine the effects of perturbations on food webs. Long-term background enrichment in ^15^N values [45] associated with increasing human development of the watershed [46] offers another possible explanation for the enrichment of ^15^N values in postdreissenid benthos, although the levels of ^15^N enrichment seen in sediment cores since dreissenid establishment in the lake [45] are unlikely to account for the full magnitude of enrichment seen in our benthos samples.

Several caveats should be mentioned. Firstly, empirical models of invertebrate production offer only general approximations of actual production rates [47], so our secondary production results should be recognized as estimates rather than direct measurements. Secondly, we assumed that dreissenid establishment was the only major change that impacted the ecology of Lake Simcoe between 1993 and 2008. There is evidence that phosphorus loading into the lake has declined by 20–30% from the mid 1990’s to the late 2000’s [46], [48], but it is unlikely that this relatively modest reduction could account for the observed changes to the food web, and we believe that dreissenid invasion offers the most parsimonious explanation for our results. Finally, our observations apply to the rocky nearshore of a relatively clear lake with an extensive littoral zone. We predict that in deeper portions of lakes, more turbid lakes, or lakes with a steeper morphometry, dreissenid enhancement of the benthic food web might be driven to a greater extent by redirection of sestonic material, and to a lesser extent by stimulation of littoral production than in our study system.

This is the first study to combine SIA with biomass and production estimates to describe how dreissenids affect food web structure and energy sources in lakes. Our findings are consistent with the hypothesis that dreissenids redirect energy and material from the water column to the littoral areas of lakes [13], [14]. By providing increased habitat availability dreissenids create a physical “matrix” for enhancements in the abundance, biomass, and production of the littoral benthos. By changing the rates and sources of energy supply to the benthos, dreissenids modify the energy base and structure of the littoral food web. Biodeposition of sestonic material by dreissenid mussels creates a direct energy link from the water column to the littoral benthos and increases the amount of energy available to detritivores and other members of the benthic community. Increased water clarity and dreissenid remineralization of sestonic-derived nutrients in the nearshore creates an indirect energy link to the nearshore by stimulating benthic littoral primary production. The redirection of production from the sestonic realm to the nearshore by dreissenids has major implications for aquatic ecosystems and may explain some of the changes in the distribution, energy sources, and production of nearshore and offshore fish communities [49], [50], nutrient cycling patterns [51], [52], and transfer of contaminants through food webs [53], [54] seen following dreissenid establishment.

## Supporting Information

Text S1 **Length-dry weight relationships used to estimate invertebrate biomass, methods used to construct length-dry weight relationships, and references for those relationships obtained from the literature.**
(DOC)
