# Supplementary material for: Invasive Mussels Alter the Littoral Food Web of a Large Lake: Stable Isotopes Reveal Drastic Shifts in Sources and Flow of Energy
Source: PLoS One. 2012 Dec 17;7(12):e51249. doi: 10.1371/journal.pone.0051249 (PMC3524176; doi:10.1371/journal.pone.0051249)
Supplement: Text S1 — Length-dry weight relationships used to estimate invertebrate biomass, methods used to construct length-dry weight relationships, and references for those relationships obtained from the literature. (DOC) [file pone.0051249.s001.doc]

**Text S1: Length-dry weight relationships used to estimate invertebrate biomass, methods used to construct length-dry weight relationships, and references for those relationships obtained from the literature.**

We used length-dry weight relationships and measurements of size frequency distributions to estimate dry mass of various benthic taxa. Where sufficient material was available we derived system-specific length-weight relationships (see table below). For taxa for which we did not have sufficient material, weight-length relationships from the literature were used. The organisms used to construct weight-length relationships were collected in Lake Simcoe using kick and sweep and airlift sampling at McRae Point and Blackbird Point in 2007, 2008, and 2009, and were stored frozen until processing. The only exception were oligochaetes which were fixed in 10% formalin and stored in ethanol prior to use in constructing length-weight relationships. Preservation of invertebrates is reported to result in reduced dry weights [1], [2], so our length-weight relationships for Oligochaeta are conservative.

Organisms used to construct length-weight relationships were photographed and measured to the nearest 0.01 mm using NIS-Elements 3.0 BR (Nikon) software on a Nikon SMZ 1500 microscope. After measurement organisms were dried at 60ºC for 24 hours, and weighed on a XS105 DualRange Analytical Balance (Mettler Toledo) to the nearest 0.01 mg. Gastropods were carefully extracted from their shells prior to weighing. Dry weight (W, mg) was plotted against length (L, mm) and a power relationship of the form W=*a*L*^b^* was fitted to the data in SigmaPlot 11.0 (Systat Software, Chicago, Illinois). Taxa names, length parameter measured, number of individuals used to construct relationships, *a*, *b* and the associated standard error, *r*^2^ and standard error of the estimate (SEE) for length-weight relationships are presented below. For length-weight relationships from the literature the source of the relationship is given.

| Taxon | Measure | *n* | *a* ± 1 SE | *b* ± 1 SE | *r*^2^ | SEE | source |
| --- | --- | --- | --- | --- | --- | --- | --- |
| **Amphipods** |  |  |  |  |  |  |  |
| *Echinogammarus ischnus* | body length | 40 | 0.0018 ± 0.0006 | 2.9162 ± 0.1781 | 0.91 | 0.0948 | this study |
| *Gammarus fasciatus* | body length | 39 | 0.0018 ± 0.0007 | 2.9710 ± 0.1743 | 0.93 | 0.1357 | this study |
| *Hyalella azteca* | body length | 43 | 0.0078 ± 0.0039 | 2.1634 ± 0.2995 | 0.62 | 0.0922 | this study |
| *Crangonyx* sp. | body length | 30 | 0.0058 ± 0.0017 | 2.798 ± 0.142 | 0.85 | n/a | 3 |
| **Isopods** |  |  |  |  |  |  |  |
| *Caecidotea racovitzai* | body length | 59 | 0.0300 ± 0.0083 | 1.6296 ± 0.1784 | 0.61 | 0.0749 | this study |
| **Decapods** |  |  |  |  |  |  |  |
| *Orconectes propinquus* | carapace length | 40 | 0.2393 ± 0.1703 | 2.7059 ± 0.2096 | 0.81 | 385.41 | this study |
| *Orconectes rusticus* | carapace length | 39 | 0.1677 ± 0.1912 | 2.8371 ± 0.3103 | 0.76 | 1164.48 | this study |
| *Orconectes virilis* | carapace length | 32 | 0.04290 ± 0.0385 | 3.1572 ± 0.2381 | 0.90 | 830.42 | this study |
| **Gastropods** |  |  |  |  |  |  |  |
| Hydrobiidae | shell height | 28 | 0.0376 ± 0.0131 | 2.3782 ± 0.2393 | 0.93 | 0.1480 | this study |
| *Physa* sp. | shell height | 22 | 0.0217 ± 0.0157 | 2.5767 ± 0.3728 | 0.83 | 0.5658 | this study |
| Pleuroceridae | shell height | 28 | 0.0134 ± 0.0165 | 2.5841 ± 0.4132 | 0.76 | 7.4408 | this study |
| **Bivalves** |  |  |  |  |  |  |  |
| Sphaeriidae | shell length | 104 | 0.0163 ± 0.0013 | 2.477 ± 0.187 | 0.87 | n/a | 3 |
| *Dreissena* spp. | shell length | 73 | 0.0014 | 2.31 | 0.92 | n/a | 4 |
| **Insects** |  |  |  |  |  |  |  |
| Elmidae | body length | 39 | 0.0008 ± 0.0009 | 3.3009 ± 0.5225 | 0.72 | 0.1945 | this study |
| Polycentropodidae | body length | 25 | 0.0071 ± 0.0015 | 2.531 ± 0.647 | 0.62 | n/a | 3 |
|  |  |  |  |  |  |  |  |
| Heptageniidae | head width | 45 | 0.1099 ± 0.0211 | 3.569 ± 0.318 | 0.90 | n/a | 3 |
| Chironomids | body length | 64 | 0.0004 ± 0.0002 | 3.0895 ± 0.2474 | 0.79 | 0.0967 | this study |
| **Worms** |  |  |  |  |  |  |  |
| Oligochaeta | body length | 29 | 0.0001 ± 0.0000 | 3.1615 ± 0.0179 | 0.99 | 0.1171 | this study |
| Flatworms | No weight-length relationship. Used average weight/ individual (0.3 mg ± 0.1 SD, *n*=18) | | | | | | |

References:

1. Leuven RSE, Brock TCM, van Druten HAM (1985) Effects of preservation on dry- and ash-free dry weight biomass of some common aquatic macro-invertebrates. Hydrobiologia 127: 151–159.
2. Gaston GR, Bartlett JHW, McAllister AP, Heard RW (1996) Biomass variations of estuarine macrobenthos preserved in ethanol and formalin. Estuaries 19: 647–679.
3. Benke AC, Huryn AD, Smock LA, Wallace JB (1999) Length-mass relationships for freshwater macroinvertebrates in North America with particular reference to the southeastern United States. J N Am Benthol Soc 18: 308–343.
4. Ozersky T, Barton DR, Depew DC, Hecky RE, Guildford SJ (2011) Effects of water movement on the distribution of invasive dreissenid mussels in Lake Simcoe, Ontario. J Great Lakes Res 37(Suppl. 3): 46–54.
